# SPDEv3.0: A multidisciplinary integrated data analysis platform

**DOI:** 10.1093/plphys/kiaf537

**Published:** 2025-10-23

**Authors:** Dong Xu, Kangming Jin, Quanling Zhang, Xianjia Zhao, Yanchun Li, Tingkai Wu, Xiaobo Wang, Yuan Yuan, Zewei An, Zhi Deng, Wenguan Wu, Han Cheng

**Affiliations:** State Key Laboratory of Tropical Crop Breeding, Rubber Research Institute, Chinese Academy of Tropical Agricultural Sciences, Haikou 571101, China; Sanya Research Institute, Chinese Academy of Tropical Agricultural Sciences, Sanya 572024, China; State Key Laboratory of Plant Environmental Resilience, College of Life Sciences, Zhejiang University, Hangzhou 310058, China; Shenzhen Branch, Guangdong Laboratory of Lingnan Modern Agriculture, Genome Analysis Laboratory of the Ministry of Agriculture and Rural Affairs, Agricultural Genomics Institute at Shenzhen, Chinese Academy of Agricultural Sciences, Shenzhen 518120, China; Bio-X Institutes, Key Laboratory for the Genetics of Developmental and Neuropsychiatric Disorders, Ministry of Education, Shanghai Jiao Tong University, Shanghai 200240, China; Shenzhen Branch, Guangdong Laboratory of Lingnan Modern Agriculture, Genome Analysis Laboratory of the Ministry of Agriculture and Rural Affairs, Agricultural Genomics Institute at Shenzhen, Chinese Academy of Agricultural Sciences, Shenzhen 518120, China; State Key Laboratory of Tropical Crop Breeding, Rubber Research Institute, Chinese Academy of Tropical Agricultural Sciences, Haikou 571101, China; Sanya Research Institute, Chinese Academy of Tropical Agricultural Sciences, Sanya 572024, China; State Key Laboratory of Tropical Crop Breeding, Rubber Research Institute, Chinese Academy of Tropical Agricultural Sciences, Haikou 571101, China; Sanya Research Institute, Chinese Academy of Tropical Agricultural Sciences, Sanya 572024, China; State Key Laboratory of Tropical Crop Breeding, Rubber Research Institute, Chinese Academy of Tropical Agricultural Sciences, Haikou 571101, China; Sanya Research Institute, Chinese Academy of Tropical Agricultural Sciences, Sanya 572024, China; State Key Laboratory of Tropical Crop Breeding, Rubber Research Institute, Chinese Academy of Tropical Agricultural Sciences, Haikou 571101, China; Sanya Research Institute, Chinese Academy of Tropical Agricultural Sciences, Sanya 572024, China; State Key Laboratory of Tropical Crop Breeding, Rubber Research Institute, Chinese Academy of Tropical Agricultural Sciences, Haikou 571101, China; Sanya Research Institute, Chinese Academy of Tropical Agricultural Sciences, Sanya 572024, China; State Key Laboratory of Tropical Crop Breeding, Rubber Research Institute, Chinese Academy of Tropical Agricultural Sciences, Haikou 571101, China; Sanya Research Institute, Chinese Academy of Tropical Agricultural Sciences, Sanya 572024, China; State Key Laboratory of Tropical Crop Breeding, Rubber Research Institute, Chinese Academy of Tropical Agricultural Sciences, Haikou 571101, China; Sanya Research Institute, Chinese Academy of Tropical Agricultural Sciences, Sanya 572024, China

## Abstract

Plant research faces persistent challenges in integrating heterogeneous data and ensuring compatibility across diverse analytical pipelines. The limited automation of existing tools often results in fragmented workflows, reducing analytical efficiency and reproducibility. To address these limitations, we developed SPDEv3.0 as a fully integrated bioinformatics platform with a graphical user interface, consolidating more than 130 functions across seven logically connected modules. SPDEv3.0 streamlines key tasks such as gene family identification, primer design, and genome feature extraction through high-level automation, enabling users to perform complex bioinformatic workflows with minimal manual intervention. For instance, collinearity analysis between *Arabidopsis thaliana* and *A. halleri* can be completed in under 2 min using only genome and annotation files, with all intermediate steps executed automatically. In addition, SPDEv3.0 offers over 30 customizable visualization functions, including genomic feature mapping, domain structure illustration, heatmaps, Circos plots, and statistical charting, all designed to help users generate publication-ready figures without coding. A total of 40 classical breeding methods have been implemented in SPDEv3.0, all of which support automated computation following user data input. Benchmarking across a range of plant genomes (including those exceeding 14 Gb) demonstrates SPDEv3.0's robust performance, scalability, and practical utility. By integrating high automation, module interoperability, and extensive visualization capacity, SPDEv3.0 provides a powerful and accessible solution for modern plant genomics and breeding research.

## Teaser

SPDEv3.0 is a novel, automated and integrative bioinformatics tool that unifies >130 functions, streamlining genomics and breeding research with unprecedented efficiency and reproducibility.

## Introduction

Recent advances in plant science have catalyzed a shift toward multidisciplinary research that integrates breeding science, molecular biology, genomics, and comparative genomics to address increasingly complex biological questions (Wang et al. 2024a; Yang et al. 2024). This transition is largely driven by the need to elucidate the genetic basis of agronomic traits, decipher evolutionary relationships, and improve crop performance under changing environmental conditions (Lyu et al. 2024). However, the integration of diverse data types and analytical workflows poses significant challenges, particularly in terms of data heterogeneity, process automation, and accessibility for researchers with varying degrees of computational expertise (Rose et al. 2021; Luo et al. 2024).

While numerous software tools exist for specific analytical tasks (He et al. 2024), most fall short in supporting comprehensive, multidisciplinary analyses within a unified framework. Researchers often face challenges such as incompatible file formats, fragmented workflows, and frequent transitions between platforms, all of which compromise efficiency and increase the likelihood of errors. Although general-purpose tools like R and SAS widely adopted for statistical analysis, especially in fields such as plant breeding (Wang et al. 2024c), they typically require a high level of statistical and programming proficiency. This steep learning curve can pose significant challenges for researchers focused more on biological interpretation than technical implementation. Core procedures, including heritability estimation, factorial analysis of variance (ANOVA), and post hoc testing, typically require several distinct steps, adding to the time and complexity of the analysis. The lack of domain-specific platforms capable of encapsulating these steps into streamlined, user-friendly workflows continue to limit accessibility and reproducibility in routine breeding studies. Similarly, genomic and comparative genomic analyses (e.g. collinearity detection) demand complex, multi-step pipelines involving sequence extraction, alignment, synteny block identification, and visualization. Integrating these functions within a cohesive environment not only enhances analytical throughput but also minimizes file compatibility issues between tools (Bernasconi 2021).

To overcome existing challenges in bioinformatics analysis, we developed SPDEv3.0, a highly integrated and automated platform designed to streamline workflows across molecular biology, genomics, and plant breeding. Compared with existing tools, SPDEv3.0 introduces three key innovations: (i) The platform offers enhanced automation for complex tasks like gene family identification and genome collinearity analysis, significantly reducing manual input. It also maintains stable performance when handling ultra-large genomes exceeding 14 Gb; (ii) a comprehensive plant breeding statistical toolkit—SPDEv3.0 is a user-friendly platform that integrates over 40 classical statistical methods, including heritability analysis, genetic correlation, and orthogonal designs, enabling breeders to perform advanced computations without relying on external software like SAS; and (iii) SPDEv3.0 also features an extensive and modular visualization suite, supporting over 20 commonly used plot types, from standard statistical graphs to complex biological diagrams (e.g. principal component analysis (PCA), violin plots) to gene family architecture diagrams and genome-scale visualizations such as *k*-mer distribution, collinearity maps, and Circos plots. These visual outputs are tightly integrated with upstream analyses and are fully customizable through an intuitive graphical interface, facilitating the generation of high-quality, publication-ready figures. Together, these advances position SPDEv3.0 as a user-friendly and high-performance solution for a wide range of genomic research applications.

## Results

### Overview of SPDEv3.0

**Figure 1.**
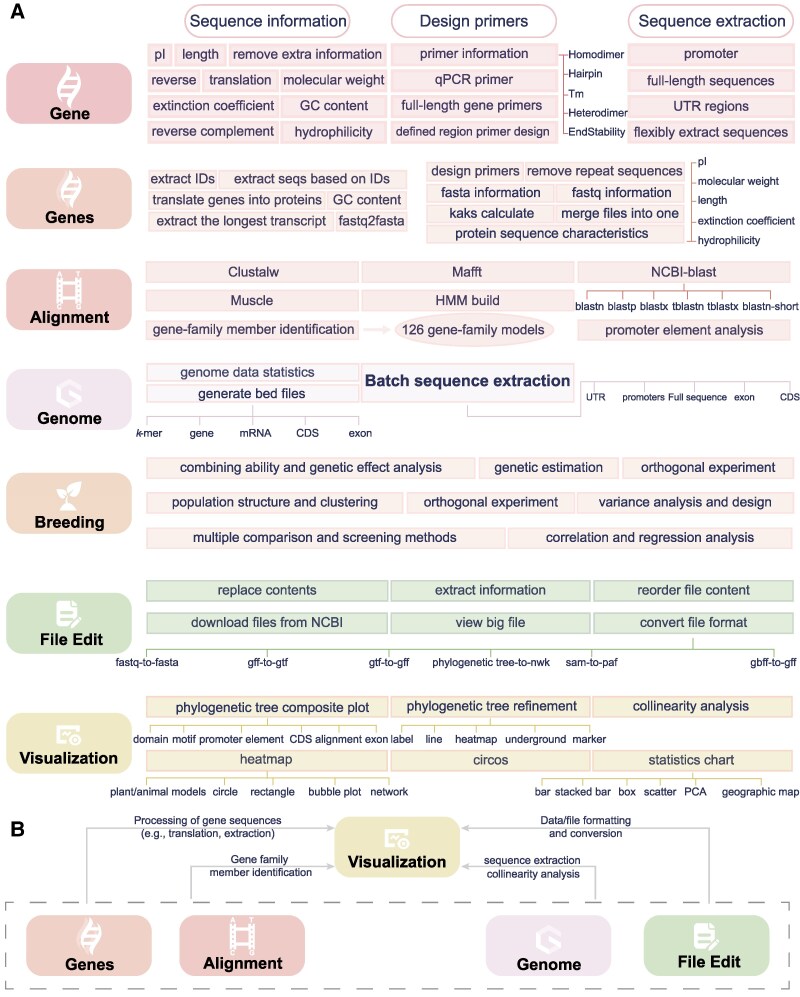
Overview of all SPDE functions. **A)** Functional allocation across modules. **B)** Integration across modules. pI, Isoelectric Point; qPCR, Quantitative Polymerase Chain Reaction; Tm, Melting Temperature; UTR, Untranslated Region; NCBI, National Center for Biotechnology Information; HMM, Hidden Markov Model; CDS, Coding Sequence; gff, General Feature Format; gtf, Gene Transfer Format; sam, Sequence Alignment/Map Format; paf, Pairwise Alignment Format; gbff, GenBank Flat File Format; PCA, Principal Component Analysis.

SPDE encompasses more than 130 functions, covering areas such as data analysis, file processing, statistical analysis, sequence preprocessing, and result visualization. These functions are systematically organized into seven modules. An overview of the primary functions within each module is presented below (Fig. 1A).

The Gene module enables users to retrieve fundamental sequence properties and to extract gene-associated regions such as promoters and untranslated regions (UTRs) directly from the genome. For primer design, the platform supports both qPCR (Quantitative Real-Time Polymerase Chain Reaction) primers and full-length gene primers, with customizable constraints on the target amplification regions. The Genes module is tailored for high-throughput applications, offering batch processing capabilities for sequence extraction, primer design, and gene translation. Additionally, it supports the identification of the longest transcript isoforms and provides basic statistical summaries for FASTA and FASTQ files. The Alignment module integrates tools such as NCBI-BLAST (Camacho et al. 2009), Diamond (Buchfink et al. 2015), ClustalW (Thompson et al. 2003), MAFFT (Katoh et al. 2005), and MUSCLE (Edgar 2004), all accessible through a user-friendly interface without requiring command-line input. Furthermore, SPDEv3.0 enables automated gene family identification and visualization using profile Hidden Markov Models (HMMs). It seamlessly integrates domain-based homology search, sequence extraction, and structural domain visualization into a streamlined workflow. Relevant HMM models can be either user-defined or selected from curated libraries (details in the section of “Several Cases-Gene Family Structural Analysis”).

The Genome module enables batch extraction of promoters, full-length genes, UTRs, and coding sequences (CDSs), and supports BED file generation for visualizing genomic features such as *k*-mers and gene distributions. The Breeding module incorporates 40 classical statistical methods, with automated execution upon data input. The File Edit module provides tools for content replacement, data restructuring, format conversion, and large-file previewing. To streamline data presentation, the Visualization module offers over 30 commonly used charting functions through an intuitive interface. Key procedures such as data normalization, significance testing, and labeling are automated, and 1,035 pre-configured color schemes are included to support high-quality, publication-ready graphics.

It is important to note that the functional modules within SPDEv3.0 are not isolated; instead, they are designed to work in an integrated and complementary manner. This modular interconnectivity is centered around the Visualization module, which serves as the primary interface for presenting analytical results across diverse biological contexts (Fig. 1B). The visualization functions can be broadly classified into three major categories: gene family visualization, genomic feature visualization, and statistical plotting. These categories are supported by input files generated from upstream modules. For example, gene family visualization depends on upstream tasks such as member identification, sequence extraction, and protein translation, which are carried out within the Alignment and Genes modules. Similarly, genome structure and feature distribution plots are supported by outputs generated from the Genome module, which enables the extraction of promoter, CDS, and UTR sequences, as well as element distribution profiling such as *k*-mers and repetitive sequences. Certain visualization types require input files with specific formats. To meet these formatting constraints, SPDEv3.0 provides the File Edit module, which allows users to manipulate, reformat, or reorganize data files accordingly. This ensures that input data are compatible with downstream visualization tools without the need for external software. Together, these interconnected modules enable SPDEv3.0 to provide a seamless, end-to-end solution for common bioinformatics analyses. From raw data processing to publication-ready figure generation, users can complete most workflows entirely within a single software environment.

### Key features and improvements in SPDEv3.0

#### 1. Automated computational methods for plant breeding

Despite advancements in molecular biology and transgenic technologies, crop improvement continues to rely heavily on classical breeding methods. Breeders typically evaluate trait heritability and genetic stability through statistical analysis of phenotypic data. Although platforms such as SAS and R offer robust tools for these analyses, their effective use often requires substantial expertise in statistics and programming, posing a barrier for some researchers.

To complement existing platforms and enhance computational accessibility, SPDEv3.0 integrates a comprehensive suite of 40 classical statistical methods frequently used in plant breeding (detailed in Supplementary Table S1), grounded in the core principles of quantitative genetics and breeding science (Chahal and Gosal 2002; Acquaah 2009). Each method is accompanied by example datasets to support ease of use, allowing users to replace the sample data with their own and execute analyses through a streamlined, one-click graphical interface. In addition, selected methods include integrated visualization tools, enabling users to interpret results immediately without requiring external plotting libraries or coding knowledge.

#### 2. Streamlining processes for efficient genomic analysis

Genomic analyses often require complex, multi-step workflows involving multiple tools and file formats. For example, collinearity and gene family analyses typically require multiple steps, including CDS extraction, sequence alignment, domain detection, and visualization, each step relying on different programs and data structures. These fragmented processes not only increase the workload but also introduce risks of format incompatibility and manual error, reducing overall analytical efficiency.

SPDEv3.0 addresses these challenges by integrating and automating key steps within a single platform, thereby enhancing both usability and reproducibility. Complex analyses can now be executed via one-click operations, minimizing manual input while maintaining analytical rigor. For instance, in collinearity analysis, SPDEv3.0 automatically extracts coding sequences from genome and General Feature Format (GFF) files, performs translation and multi-species alignment, identifies syntenic blocks, and generates publication-ready visualizations. This fully automated pipeline allows users to complete collinearity analysis between *A. thaliana* and *A. halleri* in under 2 min (Supplementary Video S1).

A similar level of automation has been implemented for gene family analysis. Unlike traditional workflows that require separate tools for domain search, member extraction, and visualization (Xu et al. 2022), SPDEv3.0 integrates these functions into a single, cohesive pipeline. Leveraging a built-in library of 126 family-specific HMM profiles, the platform performs automatic identification, sequence retrieval, conserved domain annotation, and structural visualization in one step. As an example, the identification and structural analysis of the maize *auxin response factor* (*ARF*) gene family, including domain visualization, can be completed in under 3 min (Supplementary Video S2). This integrated approach significantly enhances the speed and accuracy of gene family studies while eliminating the need for extensive file conversions or external software.

#### 3. Batch processing enhances analytical efficiency

During gene family analysis and candidate gene screening, it is often necessary to design primers for large numbers of genes to evaluate their expression patterns across different tissues or under various experimental treatments. However, designing primers for multiple genes is a complex and time-consuming process. To address this challenge, SPDEv3.0 integrates a batch primer design function that automates the process with high efficiency. Functional testing using 1,000 Arabidopsis thaliana genes demonstrated that SPDEv3.0 can complete primer design for all targets in approximately 33 s (Supplementary Video S3), substantially reducing analysis time.

Moreover, many downstream analyses depend on accurate extraction of gene-associated sequences from the genome. To support these analyses, SPDEv3.0 offers a batch sequence extraction function that retrieves various sequence types, such as full-length genes, CDS, non-coding regions, exons, and promoters, across the entire genome. For example, using the *A. thaliana* genome, the extraction of CDS for all annotated genes was completed in under 4 s (Supplementary Video S4). This functionality markedly enhances the speed and scalability of sequence-level analyses, particularly in large-scale comparative or functional genomic studies.

#### 4. Improvements in data analysis and operational convenience

Significance testing and data normalization are fundamental components of rigorous scientific analysis, particularly when dealing with datasets exhibiting substantial variability. Traditionally, these procedures have been carried out manually, often requiring extensive time and effort. In SPDEv3.0, these critical steps have been fully streamlined, markedly improving both analytical accuracy and operational efficiency. To further address the inherent complexity of biological data analysis, SPDEv3.0 incorporates a comprehensive suite of commonly used statistical and visualization tools within its integrated Visualization module. This consolidation enables seamless execution of multi-step workflows, substantially accelerating the analytical process while minimizing the need for manual intervention.

SPDEv3.0 is equipped with an intuitive, user-friendly graphical interface that supports drag-and-drop functionality and is available in both Chinese and English, thereby enhancing accessibility for a global user base. To facilitate rapid user onboarding, the platform also provides detailed user manuals and a wide array of example datasets. These settings collectively lower technical barriers and promote broader adoption of the software across diverse research communities.

### Performance and accuracy evaluation of SPDEv3.0

SPDEv3.0 simplifies complex genomic analyses by significantly reducing the need for manual input. To evaluate its computational performance and analytical accuracy, we benchmarked two core functions: synteny block detection and gene family identification. Five genome pairs were selected to represent a broad range of genome sizes and evolutionary relationships: *A. thaliana* vs. *A. lyrata*, *Populus trichocarpa* vs. *Salix babylonica*, *Oryza sativa* vs. *Zea mays*, *Glycine max* vs. *Vigna radiata*, and *Solanum lycopersicum* vs. *Vitis vinifera*. Genomic sequences and annotations were retrieved from the NCBI RefSeq database.

**Figure 2.**
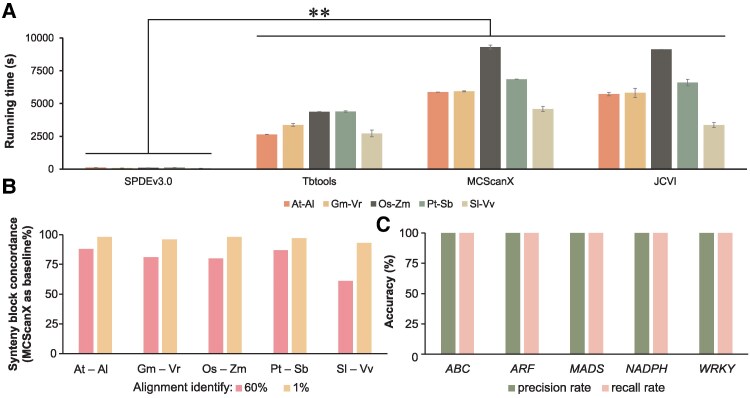
Benchmark evaluation of SPDEv3.0. **A)** Comparison of runtime (in seconds) for synteny block detection across five representative genome pairs using SPDEv3.0, TBtools, MCScanX, and JCVI. SPDEv3.0 demonstrated significantly faster performance across all datasets. At, *A. thaliana* (115 mb); Al, *A. lyrata* (199 mb); Pt, *P. trichocarpa* (378 mb); Sb, *S. babylonica* (369 mb); Os, *O. sativa* (373 mb); Zm, *Z. mays* (2.05 Gb); Gm, *G. max* (945 mb); Vr, *V. radiata* (448 mb); Sl, *S. lycopersicum* (804 mb); Vv, *V. vinifera* (478 mb). Error bars represent the standard deviation (SD) of three independent replicates. Statistical significance was evaluated using t-tests. **, *P*-value ≤0.01. **B)** Concordance of collinear gene pairs (among ten species) between SPDEv3.0 and MCScanX under two alignment identity thresholds (default: 60%, relaxed: 1%). Results show high overlap, indicating strong consistency. **C)** Accuracy of gene family member identification by SPDEv3.0 using benchmark datasets consisting of validated family members and unrelated sequences. Across all five gene families tested, SPDEv3.0 achieved 100% precision and 100% recall. *ABC*, ATP-binding cassette; *ARF*, auxin response factor; *MADS*, MADS-box transcription factor; *NADPH*, NAD(P)H dehydrogenase; *WRKY*, WRKY transcription factor. Each family contained more than 33 validated members.

In terms of runtime, SPDEv3.0 demonstrated a clear advantage over existing tools including MCScanX (Wang et al. 2024b), TBtools (Chen et al. 2020), and JCVI (Tang et al. 2024). Across all tested genome pairs, it completed collinearity analysis in an average of 89.0 s. By contrast, TBtools, MCScanX, and JCVI required 3,697.0, 5,734.0, and 5,578.6 s, respectively (Fig. 2A). One-way ANOVA confirmed that this performance difference was highly significant (*P*-value < 0.01), supporting the reproducibility and robustness of SPDEv3.0 under a standardized setup.

Since no experimentally validated gold standard exists for genome-wide synteny block detection, MCScanX was adopted as a community-recognized reference to evaluate collinearity consistency. At the default alignment identity threshold (60%), the proportion of collinear gene pairs identified by SPDEv3.0 that were also reported by MCScanX ranged from 0.80 to 0.88. When the threshold was relaxed to 1%, this value increased to over 93% across all five genome pairs (Fig. 2B). These findings reflect both the precision and adaptability of SPDE's collinearity workflow, with adjustable parameters including sequence identity, gap penalty, and minimum block size.

To further assess the functional accuracy of SPDEv3.0, we evaluated gene family member identification using five well-curated families: *ATP-binding cassette* (*ABC*), *ARF*, MADS-box transcription factor (*MADS*), *NAD(P)H dehydrogenase* (*NADPH*), and *WRKY* transcription factor (*WRKY*). Validated sequences were obtained from the UniProt reviewed protein database. A composite test dataset was created by merging these sequences with 1,000 randomly selected *A. thaliana* genes not annotated as belonging to any of the tested families. Using family-specific HMM profiles, SPDEv3.0 identified members via domain-based prediction. Across all families, SPDEv3.0 achieved 100% precision and 100% recall, correctly identifying all true family members and excluding all unrelated genes (Fig. 2C), even in the presence of complex multi-domain architectures such as those seen in the *ARF* family.

### Several cases

In addition to enhanced data processing and file handling, SPDEv3.0 introduces a significantly expanded visualization suite compared to earlier versions (Xu et al. 2021). It includes over 30 commonly used modules covering genome structure, synteny, gene expression, and statistical analysis. Visual outputs span genomic distribution plots, collinearity maps, Circos diagrams, heatmaps, expression organ maps, domain architectures, promoter motifs, and statistical charts such as volcano plots, PCA, violin plots, and Sankey diagrams.

Representative, real-world datasets were used to demonstrate key visualization functions commonly employed in plant genomics. All workflows are graphical user interface (GUI)-based, parameter-customizable, and optimized for publication-ready output. To ensure transparency and reproducibility, step-by-step instructions, intermediate summaries, and result snapshots are provided.

### Part I. Genome-level comparative and functional visualization

#### Section A. Genome structure & synteny visualization

##### Genomic feature visualization from GFF and FASTA files

SPDEv3.0 enables users to visualize genomic elements directly from GFF annotation and genome sequence files.

**Steps:**

**Figure 3.**
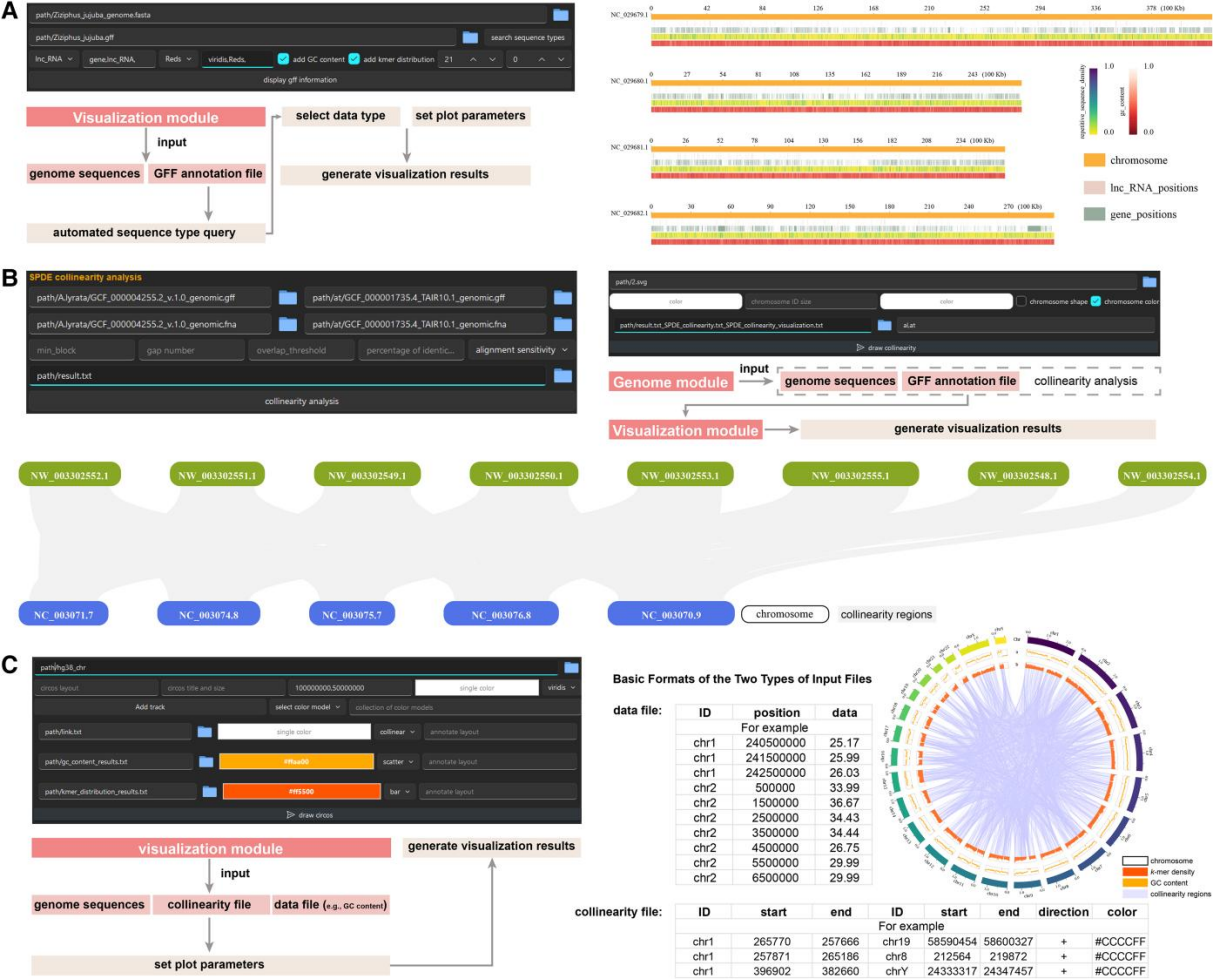
Genomic information visualization functionalities. **A)** Visualization of genomic elements (e.g. gene, lncRNA) across chromosomes using genome (FASTA) and annotation (GFF) files, with optional display of GC content and k-mer distributions. **B)** Workflow for collinearity analysis based on genome and annotation files, including CDS extraction, alignment, and synteny detection, followed by one-click visualization of syntenic relationships. **C)** Circos plot generation integrating genome structure, synteny, and statistical data. Input files include a genome file, an optional collinearity file, and user-defined data files (e.g. GC content or gene density). Representative data file formats are shown in the central panel.

1. Navigate to: Visualization module → Genome Information Plotting (Fig. 3A).
2. Load a genome sequence file (FASTA format) and its corresponding GFF annotation file.
3. Click “search sequence types” to auto-detect all available features (e.g. gene, long non-coding RNA (lncRNA)).
4. Select one or more sequence types from the dropdown menu.
5. Optionally, enable GC content or *k*-mer frequency visualization.
6. Click “Display gff Information” to generate a feature plot across chromosomes.

##### One-click collinearity analysis and visualization

SPDEv3.0 supports fully automated detection and visualization of syntenic blocks using only genome sequence and annotation files as input.

**Steps:**

1. Navigate to: Genome → Collinearity Analysis and Visualization (Fig. 3B).
2. Upload genome FASTA and GFF annotation files for each species involved.
3. Click “collinearity analysis” to automatically perform CDS extraction, protein translation, homology alignment, and synteny block detection.
4. Once the collinearity file is generated, proceed to the Visualization module.
5. Enter the species names and click “draw collinearity” to produce a visual representation of the syntenic relationships.

##### Circos plotting for integrated genomic features

SPDEv3.0 supports multi-track Circos-style plots combining sequence, synteny, and statistical data.

**Steps:**

1. Navigate to: Visualization → Circos Plot.
2. Upload required input files:
  Genome sequence file (required)
  Collinearity file (optional)
  Data file (optional. e.g. GC content, gene density)
3. Click “Add Track” to configure visual elements (shape, color, track type).
4. Click “Draw Circos” to generate the final plot (Fig. 3C).

SPDEv3.0 requires three core input files for genome visualization. The genome file defines chromosomal structure and serves as the visualization scaffold. The collinearity file encodes syntenic relationships between or within species, following a standardized format (Fig. 3C). Additional genomic features, including GC content, gene density, and k-mer distributions, are provided in tab-delimited files, as illustrated in Fig. 3C. This modular input design allows flexible integration of diverse genomic information. Most input files can be automatically generated using SPDEv3.0's Genome module.

#### Section B. Gene family structural analysis

SPDEv3.0 enables automated identification of gene family members using HMM profiles, and supports visualization of conserved domains, motifs, promoter elements, and phylogenetic trees.

**Steps:**

**Figure 4.**
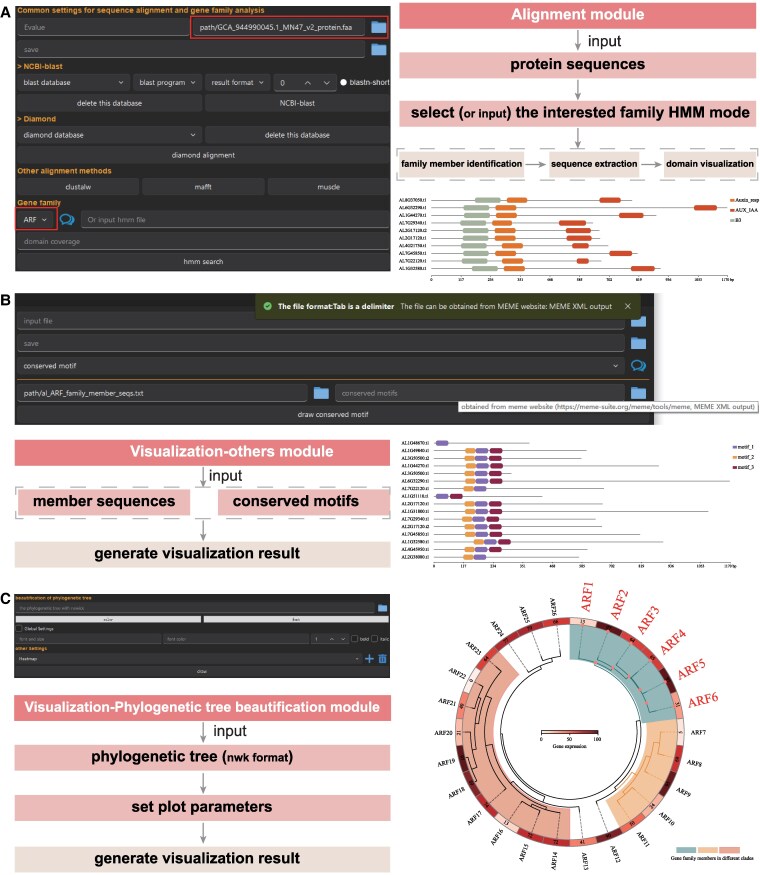
Gene family analysis and visualization features. **A)** Gene family members are identified and domain structures visualized by inputting protein sequences and applying either built-in or user-defined HMM profiles (as highlighted in red boxes). **B)** Motif data from MEME website is integrated with customizable visualization. Tooltip-based guidance appears upon clicking or hovering over interface elements. **C)** Phylogenetic tree beautification supports nwk format input and allows for flexible customization, including branch styling, label formatting, and integration of heatmaps, markers, and labels.

1. Navigate to: Alignment → Gene Family.
2. Upload a protein FASTA file as input, then either:
  Select a predefined model from the 126 gene family HMMs integrated into SPDEv3.0, or
  Import a custom HMM file downloaded from public databases (e.g. InterPro-Pfam).
3. (Optional) To build a new HMM model, go to: Alignment module → HMM Construction, and input a set of highly conserved protein sequences. Click “Build HMM model” to generate a profile HMM automatically.
4. Click “hmm search”. SPDE will identify gene family members, extract their sequences, and generate structural domain architecture plots (Fig. 4A).
5. To visualize conserved motifs or cis-regulatory elements, export motif results from MEME Suite and promoter annotations from PlantCARE, then go to:
  Visualization → Others module, choose the desired file, and select the appropriate visualization format.
6. Tooltips and button-triggered hints are provided throughout the interface to guide users during analysis and parameter selection (Fig. 4B).
7. For phylogenetic visualization, import a tree in nwk format to Visualization module → Phylogenetic tree beautification.

Users can customize branch color, font, size, and line thickness. Additionally, SPDEv3.0 supports the integration of auxiliary elements such as heatmaps, labels, markers, and line styles to enhance the interpretability and aesthetic quality of the phylogenetic tree (Fig. 4C).

### Part II. Expression & statistical pattern visualization

SPDEv3.0 integrates a wide array of heatmap and statistical charting functions to support the visualization of complex biological data in accordance with scientific publication standards.

#### Section A. Expression & heatmap

The Visualization → Heatmap module enables flexible heatmap generation using custom data matrices.

**Steps:**

**Figure 5.**
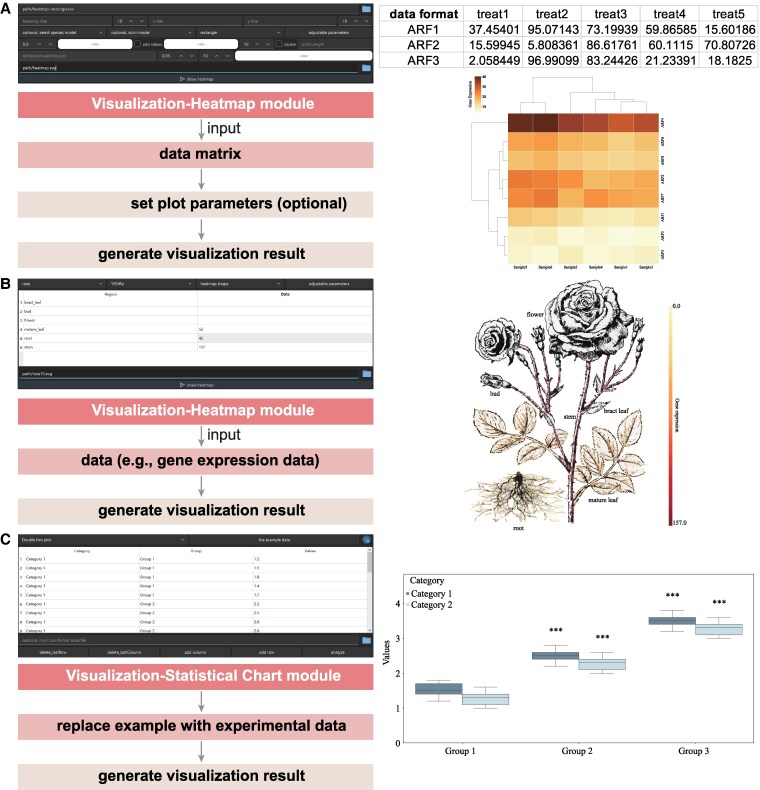
Heatmap and statistical chart visualization. **A)** Clustered rectangular heatmap generated from a gene expression matrix, illustrating SPDEv3.0's support for hierarchical clustering and dynamic color mapping. The second column displays the required input data format. **B)** Expression matrix data can be projected onto plant anatomical diagrams for organ-specific visualization. **C)** Using a box plot as an example, this panel illustrates the statistical charting process within the Visualization module, which supports automatic data normalization and significance analysis. Double boxplots show the distribution of values across different groups (Group 1, Group 2, Group 3) and categories (Category 1, dark blue; Category 2, light blue). The lower and upper edges of each box represent the first (Q1) and third quartiles (Q3), respectively, and the horizontal line within the box indicates the median. Differences between categories within each group and the reference group (Group 1) were assessed using independent-sample t-tests. *** denotes highly significant differences.

1. Navigate to: Visualization module → Heatmap (Fig. 5A).
2. Upload a matrix-style data file (see Fig. 5A for format reference).
3. Select a heatmap style: traditional hierarchical clustering, circular heatmap, or phylogeny-linked heatmap.
4. Choose from 20 pre-defined color palettes.
5. Click Draw Heatmap to generate the figure.

Additionally, users can project gene expression values directly onto plant organ models by selecting Organ Heatmap, which highlights organ-specific expression patterns with intuitive colors (Fig. 5B).

#### Section B. Statistical graph generation

The Statistical Chart functions consolidates commonly used statistical plots tailored for biological datasets.

**Steps:**

1. Navigate to: Visualization module → Statistical Chart (Fig. 5C).
2. Select the desired chart type (e.g. volcano plot, PCA, boxplot, violin plot, bar chart, bubble chart).
3. Replace the example values in the input table with your own dataset.
4. Configure chart properties such as grouping variables, axes labels, and scale options.
5. Significance testing (e.g. *P-*values) is automatically performed.
6. Click “analyze” to obtain a ready-to-publish visualization.

These functions enable users to rapidly transform raw numeric data into high-quality, interpretable figures suitable for publication or downstream analyses.

## Discussion

Compared to earlier tools that required step-by-step manual operations, SPDEv3.0 incorporates a series of design optimizations to enhance the efficiency, reproducibility, and user accessibility of biological data analysis. Central to its architecture is the full automation of steps that do not require user decision-making. For example, in collinearity analysis, users need only provide genome and annotation files; SPDEv3.0 automatically performs coding sequence extraction, protein translation, sequence alignment, collinearity block identification, and figure generation—eliminating manual intervention and reducing errors associated with file incompatibility. At the data processing level, the platform supports folder-level input and automated batch iteration, enabling one-click execution across multiple datasets. Statistical analyses are streamlined through built-in data normalization and significance testing, while visualization is optimized via automated layout adjustment and over 1,000 predefined color schemes. These features have been validated on diverse real-world datasets, confirming significant gains in analytical speed, robustness, and ease of use.

Despite the broad functionality and strong performance of SPDEv3.0, certain aspects warrant further development to ensure full scalability and versatility across genomic contexts. To date, functional validation has primarily been conducted using small to moderately sized plant genomes (e.g. *Z. mays*, ∼2 Gb). However, with the increasing availability of ultra-large genome assemblies, including those exceeding 5 Gb, it is critical to assess the platform's robustness in handling such data. SPDEv3.0 addresses potential performance bottlenecks through an integrated memory management strategy based on the pyfaidx library, which enables indexed, region-specific access to genome sequences rather than full memory loading (Shirley et al. 2015). This architecture minimizes RAM consumption during sequence operations such as CDS or promoter extraction, which are typically localized and do not require genome-wide parsing. To empirically assess scalability, we tested SPDEv3.0 on the *Allium cepa* genome, which is approximately 14 Gb in size. The GFF annotation visualization was successfully completed in under 7 min on a standard desktop computer, without memory errors or crashes (Supplementary Fig. S2). This real-world result supports the theoretical scalability of SPDEv3.0 and highlights its capability to process ultra-large genomes with high efficiency and stability. Future benchmarking will focus on evaluating its scalability across large-genome species and diverse computing environments. In addition, while the current visualization and statistical functions are optimized for structured and complete datasets, specialized applications involving sparse matrices, small-sample inference, or high-throughput phenomics may require additional customization. Upcoming versions of SPDE will prioritize enhanced data compatibility and the integration of robust algorithms to support a broader range of biological research scenarios.

Looking forward, SPDEv3.0 is designed with modular extensibility in mind, and a major direction for future development involves the integration of machine learning (ML) and deep learning (DL) models. This will include supervised models for trait prediction, unsupervised clustering for omics data mining, and graph neural networks for biological network inference. These models will be incorporated as plug-in modules linked to the existing functional framework. For example, outputs from the gene family module (e.g. domain architectures) and expression module (e.g. heatmaps) can be used as input features for classification models. Similarly, genome structural outputs such as collinearity blocks or *k*-mer distributions could be fed into DL models for pattern discovery across species or populations. In addition, we plan to establish a model training interface within SPDE that allows users to train, validate, and deploy their own ML models using curated biological datasets. To ensure ease of use, we will provide pretrained models, optimized pipelines, and guided parameterization, minimizing the technical barriers for users unfamiliar with ML frameworks. By combining automated genomic analysis with intelligent model-based interpretation, we aim to evolve SPDEv3.0 from a high-throughput data processing platform into a smart analytical assistant capable of supporting next-generation plant biology and breeding research.

## Materials and methods

### Data source

All genomic datasets used to evaluate SPDE's functionality were derived from real biological data, rather than simulated or synthetic datasets, and were primarily sourced from the NCBI RefSeq database. Similarly, Pfam annotations (Mistry et al. 2021) employed for protein domain and gene family identification were sourced from the InterPro-Pfam (https://www.ebi.ac.uk/interpro/entry/pfam/#table) database. These datasets ensure the accuracy and reliability of the analyses conducted using SPDE.

### Construction of gene family identification database

Gene family members often contain multiple protein domains, not all of which are specific to their respective families. To enable accurate and high-throughput gene family identification, we conducted an extensive literature survey and curated a set of 126 widely studied gene families (Supplementary Table S2). For each gene family, we identified and compiled its characteristic domains, defined as core conserved motifs essential for function, using HMM-based models. These curated HMM profiles were then integrated into a dedicated domain database within SPDEv3.0, facilitating automated and standardized identification of gene family members. For gene families that contain multiple conserved domains (e.g. *ARF* family, which includes both B3 and Auxin_resp domains), all relevant domain HMM profiles were integrated into a single composite model file. During gene family identification, SPDE performs independent domain searches for each HMM model and determines candidate genes based on domain-level detection. Only genes that contain all required domains are retained as final family members.

In addition to pre-integrated Pfam models, SPDE also allows users to input custom HMM models. These models can be directly downloaded from the InterPro database or constructed de novo using SPDE's built-in HMM model building function, which leverages multiple sequence alignment followed by hmmbuild to generate profile HMMs from user-provided protein sequences (refer to the section titled “Automated Construction of Profile HMMs from Protein Sequences”).

### Calculate methods

SPDEv3.0 includes default statistical methods for significance testing, such as the t-test, ANOVA, and multivariate ANOVA. For data normalization, the platform employs the z-score method as the default approach, with additional normalization techniques available for user selection.

### Python packages and software

To improve the accuracy and efficiency of data extraction and computation, SPDEv3.0 incorporates a variety of Python packages optimized for high-performance bioinformatic processing. Additionally, several Python-based frameworks were employed to support interface development and enhance the visual design and user experience of the software. A detailed list of the packages and frameworks used is provided in Supplementary Table S3.

### Transcription factor binding site prediction

Complete promoter sequences were extracted from the genomes of *A. thaliana*, *O. sativa*, *Z. mays*, and *P. trichocarpa*. These sequences were subsequently analyzed using PlantPAN 4.0 (Chow et al. 2024) to identify transcription factor binding sites (TFBSs). The detected TFBSs were classified and curated into a structured database according to their corresponding transcription factors. During subsequent analyses, SPDEv3.0 automatically selects the appropriate TFBS database based on user-defined transcription factors, conducts sequence alignment using SeqMap (Jiang and Wong 2008), and performs statistical evaluation of the binding sites. The final results are presented through integrated visualizations to facilitate downstream interpretation.

### Data visualization

To address visualization needs, we implemented a Python-based program that converts input data into SVG vector graphics, ensuring scalability and optimal clarity. For color scheme selection, we curated 1,035 color palettes from contemporary publications, allowing users to switch effortlessly between modes with a single click. This flexibility enhances the interpretability of complex datasets.

### Plant and cell models

To ensure model accuracy and provide versatile visual representation, we collected 38 widely studied plant model images and two cell models (encompassing plant and animal cells) from specialized textbooks. These images were edited using Adobe Illustrator, organizing different organs or tissues into separate layers (examples shown in Supplementary Fig. S1). Upon model selection, this feature colors various model components based on user-provided data (Fig. 5B).

### The logic and detailed algorithms of SPDEv3.0

Many routine bioinformatics analyses involve a series of standardized steps that do not require manual decision-making, making them well-suited for automation. For example, gene family analysis typically comprises member identification, sequence extraction, domain annotation, and result visualization. However, most existing tools require users to execute each step manually, which reduces overall analytical efficiency. To address this limitation, SPDEv3.0 streamlines these workflows by automating intermediate steps. In the sections that follow, we detail the underlying logic and algorithmic design principles that support key automated functions in SPDEv3.0.

### Automated extraction and primer design workflow

To enhance the specificity and amplification efficiency of primer design, we developed an automated two-round full-length primer design workflow. In the first round, according to the position of the target gene in the GFF annotation file, the pipeline extracts the 5′ and 3′ UTRs and accurately identifies the boundaries of CDS, ensuring that the designed primers are positioned within the UTRs to improve specificity. The Primer3 library of Python is employed to automatically define the primer search intervals and product size ranges, outputting multiple candidate primer pairs along with their thermodynamic characteristics, including melting temperature, GC content, self-complementarity, and hairpin potential. The second-round amplification is performed using the first-round PCR product as the template. For this stage, the primers are directly derived from the first and last 20 base pairs of the CDS to form nested internal primer pairs. Regular expression matching is used to determine the relative position of the CDS within the mRNA, and reverse complementation is applied to the 3′-end (reverse) primer to ensure synthesis compatibility. The entire design module is integrated into a graphical user interface. Memory caching and event-driven conditions are implemented to minimize redundant I/O operations, thereby improving computational efficiency (Supplementary Video S5).

### Identification of the longest representative transcript per gene

Using species CDS sequences as templates, transcript sequences are aligned and subsequently filtered based on two parameters: alignment identity (default ≥90%) and coverage (default ≥50%), both adjustable via the GUI. Pairs meeting both criteria are grouped by reference gene loci. Genes are parsed based on underscore-delimited naming conventions, accommodating different annotation formats. For each gene cluster, the transcript with the maximum length is selected as the longest representative and recorded. Additionally, transcript density statistics are calculated based on the number of matching transcripts per locus. The workflow includes automated handling of intermediate files and comprehensive error catching, ensuring robustness and reproducibility in large-scale transcriptome analyses.

### Automated construction of profile HMMs from protein sequences

To enable streamlined generation of profile HMMs, we developed an automated pipeline that integrates sequence alignment and model building. The user-provided protein sequences are first aligned using MAFFT, with selectable alignment modes: high accuracy mode invokes –localpair and –maxiterate 1,000 for iterative refinement, while fast mode uses simplified heuristics (−retree 1, –maxiterate 0) for rapid processing. The resulting alignment is saved in FASTA format and processed using the built-in hmmbuild subroutine to generate the corresponding HMM profile. The system automatically resolves output paths, defaulting to a standardized file name if not specified, and handles whitespace in file or folder names to prevent execution errors. Throughout the process, real-time user feedback is provided via GUI elements, and error handling is implemented to capture subprocess failures with descriptive warnings. This design ensures reproducibility, user configurability, and robustness, supporting efficient model construction even in large-scale or high-throughput environments.

### Automated identification and domain characterization of gene family members

To enable automated identification and structural domain analysis of gene family members, we implemented a modular workflow centered on HMM-based sequence profiling. Users may specify a custom HMM file or select from a pre-integrated gene family model database. Protein sequences are queried against the HMM using hmmsearch, with adjustable E-value thresholds (default: 1e-5) and parallel execution configured to utilize available CPU resources. Domain hits are parsed to extract coverage and completeness; only candidates with >80% domain coverage are retained for downstream analysis. For each complete-domain hit, representative sequences are aggregated and filtered to remove redundancy. The longest shared domain-bearing members are then extracted and saved for further use. Optionally, if a comprehensive Pfam database (https://github.com/simon19891216/SPDE/releases/tag/database) is present, the workflow extends to full domain annotation of family members, and outputs all structural motifs in tab-delimited files. All outputs include domain coverage reports, non-redundant member sequences, optional notes on duplicated protein entries, and protein domain visualization results. The workflow integrates real-time GUI feedback, temporary file management, and efficient data handling to facilitate gene family characterization across large datasets.

### Cross-species collinearity analysis based on homologous gene anchoring and dynamic programming

To identify conserved syntenic blocks between two species, we developed an automated collinearity analysis pipeline that integrates genome annotation parsing, CDS translation, protein alignment, and block inference. GFF and genome FASTA files for both species are used to extract and translate CDS into protein sequences. Homologous relationships are identified using Diamond -based all-against-all protein alignment, with alignment identity thresholds set by the user (default ≥60%). Gene positions are parsed from GFF files and used to map aligned gene pairs across chromosomes. Homologous pairs are clustered by chromosome and ranked by genomic coordinates. Inspired by the MCScanX algorithm, a dynamic programming approach is applied to assemble syntenic chains using match scores, gap penalties, and user-defined parameters including minimum block size, maximum allowed gene gaps, and block overlap thresholds. To avoid redundancy, overlapping blocks are filtered based on gene-pair intersection ratios. The final results are saved in two formats: a tab-delimited file compatible with MCScanX and a coordinate file suitable for collinearity visualization, with strand consistency and genomic boundaries inferred from annotation files. This pipeline ensures reproducibility, user control, and scalable detection of conserved collinear regions in large comparative genomic datasets.

### Synteny detection accuracy and performance benchmarking

Given the lack of an experimentally validated gold standard for genome-wide synteny block detection, we adopted a consensus-based comparative framework to evaluate the accuracy and runtime efficiency of SPDEv3.0. Five representative genome pairs were selected for benchmarking: *A. thaliana* vs. *A. lyrata*, *P. trichocarpa* vs. *S. babylonica*, *O. sativa* vs. *Z. mays*, *G. max* vs. *V. radiata*, and *S. lycopersicum* vs. *V. vinifera*. These species pairs span a broad range of genome sizes (from ∼115 mb to ∼2.05 Gb), cover both monocots and dicots, and represent diverse life histories (e.g. herbaceous and woody plants), thus ensuring a biologically and technically representative test set. All genome sequences and corresponding GFF annotation files were downloaded from the NCBI RefSeq database to ensure consistency in data quality.

To assess computational efficiency, collinearity analyses were performed using SPDEv3.0, MCScanX, JCVI, and TBtools under their respective default parameter settings. All tools were executed on the same hardware platform, consisting of a 64-bit Windows system with 32 GB of RAM. For SPDEv3.0, this included a default alignment identity threshold of 60% for homologous gene pairs. Runtime was recorded from the initiation of gene alignment to the generation of the final collinearity file. This standardized computational environment enabled a fair and reproducible comparison across tools.

Due to the heuristic and parameter-sensitive nature of synteny detection algorithms, we assessed relative accuracy rather than absolute correctness. Specifically, we computed the proportion of collinear gene pairs identified by SPDEv3.0 that were also recovered by MCScanX, which was used as a community-recognized reference tool rather than a definitive truth set. In addition to default settings, SPDEv3.0 analyses were repeated using a relaxed alignment identity threshold (≥1%) to evaluate its sensitivity across a broader homology spectrum.

### Accuracy evaluation of gene family identification

To evaluate the accuracy of SPDEv3.0 in gene family identification, we constructed a composite benchmark dataset composed of both positive and negative controls. The positive control consisted of experimentally validated protein sequences from five well-characterized gene families: *ABC*, *ARF*, *MADS*, *NADPH*, and *WRKY*, all sourced from the UniProt database. For each family, at least 33 representative members were randomly selected.

The negative control included 1,000 randomly selected protein-coding genes from A. thaliana that are not annotated as members of the five test families, ensuring they lack known functional domains associated with the selected Pfam models.

The combined dataset (positive + negative) was then queried using the HMM-based identification pipeline in SPDEv3.0. Prediction performance was quantified using standard classification metrics:

Precision = TP/(TP + FP), where TP (true positives) are experimentally verified sequences correctly identified as family members, and FP (false positives) are non-family sequences incorrectly identified.

Recall = TP/(TP + FN), where FN (false negatives) are experimentally verified family members that were not detected by the model.

This evaluation framework enables simultaneous assessment of both sensitivity (recall) and specificity (via precision) across gene families with varying domain architectures, including both single-domain (e.g. *ABC*, *WRKY*) and multi-domain families (e.g. *ARF*). All searches were conducted using SPDEv3.0 under default thresholds without manual post-filtering, ensuring that accuracy metrics reflect real-world, automated application scenarios.

### Demonstration of visualization functionality using multi-source biological datasets

To demonstrate the functionality and versatility of the Visualization module in SPDEv3.0, we employed real biological datasets from multiple sources. Specifically, the genome sequence and GFF annotation files of *A. cepa* were downloaded from the hortDB database (Li et al. 2024) and used to showcase genomic feature visualization based on GFF annotations. For genome-wide synteny visualization, *A. thaliana* data were retrieved from the NCBI RefSeq database. Circos plot functionality was demonstrated using the human HG38 reference genome to highlight the software's applicability beyond plant species. Gene family visualization was performed using *A. lyrata* genome data, also obtained from NCBI. In addition, gene expression data for selected genes were retrieved from FlowerBase (https://bioinformatics.hainanu.edu.cn/flowerbase/static/index.html) to support expression-related visualizations. All remaining datasets were generated in-house by our laboratory.

### Accession numbers

Sequence data from this article can be found in the GenBank/EMBL data libraries, and the relevant accession numbers in Supplementary Table S4.

## Supplementary Material

kiaf537_Supplementary_Data

## Data Availability

The executables (Windows and MacOS), example data, tutorials are all available at https://github.com/simon19891216/SPDE. Additionally, users can access a complete set of tutorials on software usage through this account.

## Supplementary data

The following materials are available in the online version of this article.

**Supplementary Figure S1.** Some examples of plant models in SPDE.

**Supplementary Figure S2.** GFF information of *A. cepa*.

**Supplementary Table S1.** Computational methods and taxonomic summary in plant breeding.

**Supplementary Table S2.** Gene family models included in SPDE.

**Supplementary Table S3.** Python packages and software utilized for SPDE.

**Supplementary Table S4.** Sequence data and the relevant NCBI accession IDs.

**Supplementary Video S1.** Collinearity analysis between *A. thaliana* and *A. halleri*.

**Supplementary Video S2.** Identification and structural analysis of the maize *ARF* gene family.

**Supplementary Video S3.** Batch design process of SPDEv3.0 primers.

**Supplementary Video S4.** Batch CDS extraction process of SPDEv3.0.

**Supplementary Video S5.** Design process of full-length gene primers in SPDEv3.0.

## Dive Curated Terms

The following phenotypic, genotypic, and functional terms are of significance to the work described in this paper:
