# Supplementary material for: SPDEv3.0: A multidisciplinary integrated data analysis platform
Source: Plant Physiol. 2025 Oct 23;199(3):kiaf537. doi: 10.1093/plphys/kiaf537 (PMC12596362; doi:10.1093/plphys/kiaf537)
Supplement: kiaf537_Supplementary_Data [file kiaf537_supplementary_data.zip › Supplementary Data.pdf]

SPDEv3.0: A Multidisciplinary Integrated Data Analysis Platform

Dong Xu[1,2#*], Kangming Jin[3*], Quanling Zhang[4*], Xianjia Zhao[5*], Yanchun Li[4*], Tingkai Wu[1,2], Xiaobo Wang[1,2], Yuan Yuan[1,2], Zewei An[1,2], Zhi Deng[1,2], Wenguan Wu[1,2], Han Cheng[1,2#]

[1]National Key Laboratory for Tropical Crop Breeding, Rubber Research Institute, Chinese Academy of Tropical Agricultural Sciences, Haikou 571101, Hainan China

[2]Sanya Research Institute, Chinese Academy of Tropical Agricultural Sciences, Sanya 572024, Hainan China

[3]State Key Laboratory of Plant Environmental Resilience, College of Life Sciences, Zhejiang University, Hangzhou 310058, China

[4]Shenzhen Branch, Guangdong Laboratory of Lingnan Modern Agriculture, Genome Analysis Laboratory of the Ministry of Agriculture and Rural Affairs, Agricultural Genomics Institute at Shenzhen, Chinese Academy of Agricultural Sciences, Shenzhen 518120, China

[5]Bio-X Institutes, Key Laboratory for the Genetics of Developmental and Neuropsychiatric Disorders, Ministry of Education, Shanghai Jiao Tong University, Shanghai 200240, China

*These authors contributed equally to this work.

#Correspondence: Han Cheng, Email: forcheng@gmail.com; Dong Xu, Email: xudongzhuanyong@163.com

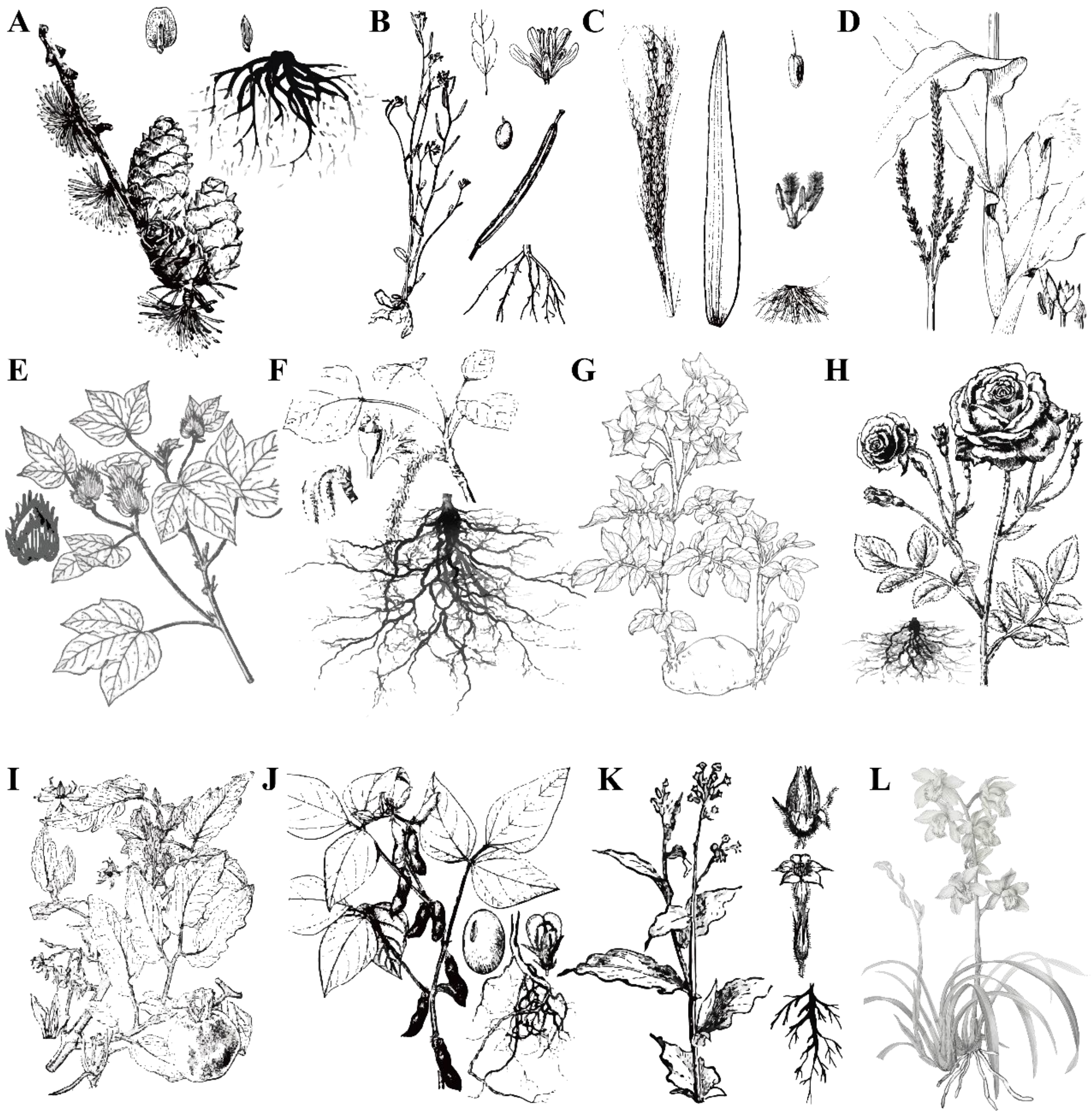

Supplementary Figure S1 Some examples of plant models in SPDE

A. pine; **B.** Arabidopsis; **C.** rice; **D.** maize; **E.** cotton; **F.** poplar; **G.** potato; **H.** rose; **I.** tomato; **J.** soybean; **K.** tobacco; **L.** orchid.

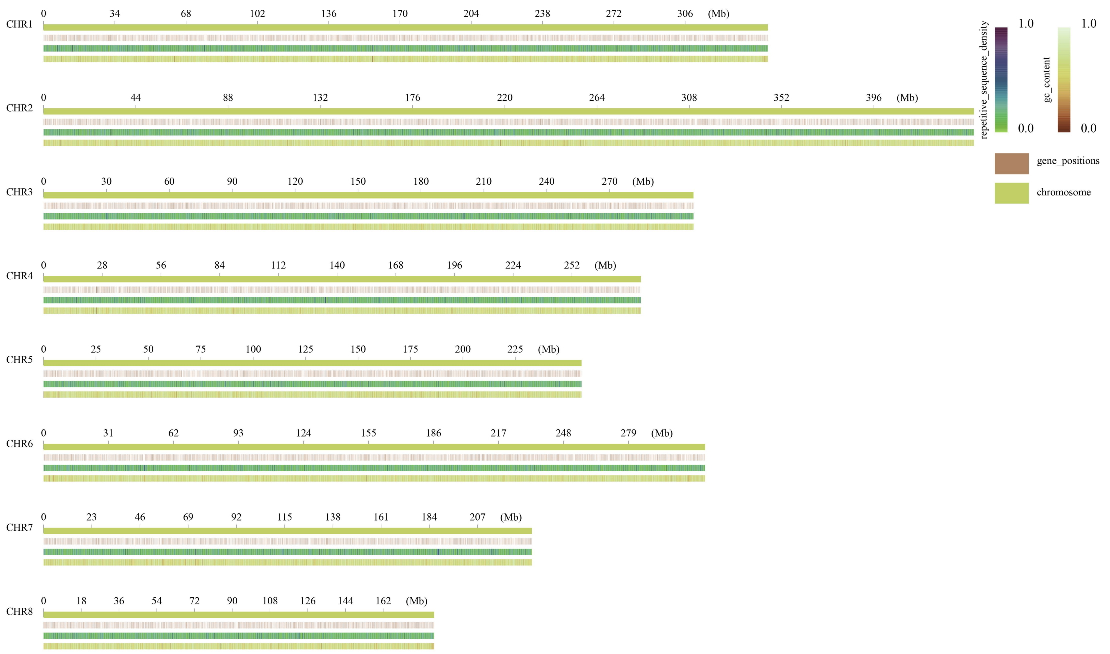

Supplementary Figure S2 GFF information of *Allium cepa*

On a standard desktop computer (32 GB RAM), the genomic data of *A. cepa* (genome size ~14 Gb) can be visualized and rendered within seven minutes.

Supplementary Table S1 Computational Methods and Taxonomic Summary in Plant Breeding

Category	Method 1	Method 2	Method 3	Method 4	Method 5	Method 6
**Variance Analysis and Design**	One-way ANOVA<br>Variance components analysis	One-way MANOVA<br>Covariance analysis	Two-factor variance analysis<br>Completely randomized design	Two-way analysis of variance<br>Split-plot design	Two-way MANOVA<br>Latin square design	Three-way randomized block design<br>Balanced incomplete block design
**Genetic Estimation**	Broad-sense heritability	Narrow-sense heritability	Heritability Estimation	Genetic correlation	Estimated variance components	
**Correlation and Regression Analysis**	Canonical correlation analysis	Multi-correlation coefficient	Partial correlation coefficient	Rank correlation coefficient	Power regression	
**Population Structure and Clustering**	Cluster analysis	Dynamic clustering method	Trait clustering analysis	Principal component analysis		
**Significance and Hypothesis Testing**	Significance analysis<br>Confidence interval estimation	Significant difference<br>Rank sum test	Paired significance test	Normality test	Variance homogeneity test	Residual analysis
**Combining Ability and Genetic Effect Analysis**	Combining ability analysis	Diallel cross combining ability				
**Multiple Comparison and Screening Methods**	Multiple comparison method	Stepwise discriminant analysis	Discriminant analysis			
**Orthogonal Experiment**	Orthogonal test analysis					

Supplementary Table S2 Gene family models included in SPDE

Abbreviation	Full term	Abbreviation	Full term
ABC1K	Activity of BC1 complex kinase	HSP90	Heat shock proteins 90
ABI5	ABA Insensitive 5	JAZ	Jasmonate ZIM-domain
AGPase	ADP-glucose pyrophosphorylase	KK	Ion transport protein
ALMT	Aluminum-activated malate transporter	LBD	Lateral organ boundaries domain
AMT	Ammonium transporters	LFY	LEAFY
AP2	APETALA2	LHC	Light-harvesting chlorophyll a/b-binding
APX	Ascorbate peroxidase	LRR	Leucine Rich Repeat
AQP	Aquaporin	LSD	LSD1 zinc finger
ARF	Auxin response factor	MAPK	Mitogen-activated protein kinases
ARR	Arabidopsis response regulator	MIKC_MADS	MIKCC-type MADS-box
AUX_IAA	Auxin/indole-3-acetic acid	M-type_MADS	M-type MADS-box transcription factor
B3	B3	MYB	MYB transcription factor
BBR-BPC	BARLEY B RECOMBINANT/BASIC PENTACYSTEINE	MYB_related	MYB-related transcription factor
BES1	BRI1-EMS-SUPPRESSOR1	NAC	NAM, ATAF1/2 and CUC2
bHLH	Basic helix-loop-helix	NADPH	NAD(P)H dehydrogenase
bZIP	Basic leucine zipper	NF-X1	Nuclear factor X1
C2H2	C2H2 zinc finger protein	NF-YA	Nuclear factor YA
C3H	C3H type zinc finger protein	NF-YB	Nuclear factor YB
CaM	Calmodulin	NF-YC	Nuclear factor YC
CAMTA	calmodulin-binding transcription activator	NRAMP	Natural resistance-associated macrophage protein
CBF_DREB	C-repeat binding factor /dehydration-responsive element bindin	NRT1	nitrate transporter 1
CBL	Calcineurin B-like	NZZ_SPL	SPOROCYTELESS/NOZZLE
CDPK	Calcium-dependent protein kinases	P450	Cytochrome P450
CesA	Cellulose synthase A	PAL	Phenylalanine ammonia lyase
CHS	Chalcone synthase	Peroxiredoxin	Peroxiredoxin
CNGC	Cyclic nucleotide-gated channel	PHT	Phosphate transporter genes
CO-like	CONSTANS	PIN	PIN-FORMED
CPP	cystein-rich polycomb-like protein	PLC	Phospholipase C
CRY	Cryptochrome	PP2C	Protein phosphatase 2C
Cyclophilin	Cyclophilin	PUB	Plant U-box
DBB	double B-box zinc finger	PYL	Pyrabactin resistance 1-like proteins
DELLA	DELLA	RAV	Related to ABI3 and VP1
Dof	DNA binding with one finger	RBOH	Plant respiratory burst oxidase homolog
E2F_DP	E2F/DP	RCC1	Regulator Of Chromosome Condensation 1
EIL	ethylene-insensitive3/Ethylene-insensitive3-like	RLK	Receptor-like kinases
ERF	ethylene responsive factor	S1Fa-like	S1fa transcription factor
ETR_EIN	Ethylene-insensitive3/ethylene-insensitive3-like	SAP	Stress associated proteins
Expansin	Expansin	SAUR	Small auxin up-regulated RNA
FAD	Fatty acid desaturase	SBP	SQUAMOSA promoter-binding protein
FAR1	far-red-impaired response	SMXL	SUPPRESSOR of MAX2 1-LIKE proteins
FGGY	FGGY carbohydrate kinase	SnRK1	SNF1/AMPK/SnRK1 serine-threonine kinase
G2-like	Golden2-like	SOD	Superoxide dismutase
GATA	GATA	SPX	SYG1/Pho81/XPR1
GCR	GC receptor	SRS	SHORT INTERNODES (SHI)-related sequence
GDH	Glutamate dehydrogenases	STAT	signal transducer and activators of transcription
GeBP	GLABROUS1 enhancer-binding protein	STP	Sugar transporter
GH3	Gretchen Hagen3	SWEET	Sugars Will Eventually be Exported Transporter
GID1	Gibberellin insensitive dwarf 1	TALE	Three Amino acid Loop Extension
GP	Glyceraldehyde-3-phosphate dehydrogenase	TCP	Teosinte branched1/Cincinnata/proliferating cell factor
GR	Glutathione reductase	TIR1_AFB	auxin receptor (TIR1/AFBs)
GRAS	GRAS	TMT	Tonoplast monosaccharide transporter
GRF	Growth-regulating factor	Trihelix	Trihelix transcription factor
GS	Glutamine synthetas	VOZ	Vascular plant one zinc-finger
GST	Glutathione S-transferase	VQ	VQ motif-containing protein
HB-other	Homeobox	WAK	Wall-associated kinase
HB-PHD	homeobox-Plant homeo-domain	WHY	Whirly
HB-ZIP	Homeobox-leucine zipper	WOX	WUSCHEL related homeobox
HD-ZIP	homeodomain-leucine zipper	WRKY	WRKY transcription factor
HGP	WD domain, G-beta repeat	XTH	Xyloglucan endotransglucosylase/hydrolase
HK	Hexokinase	YABBY	YABBY transcription factor
HRT-like	Hordeum repressor transcription-like	YUCCA	Tryptophan aminotransferases
HSF	Heat shock factor	ZF-HD	Zinc finger-homeodomain
HSP	heat shock protein	ABC	ATP-binding cassette

Table S3 Python packages and software utilized for SPDE

Category	Name	Version	Roles in SPDE
**Data Processing and Analysis**	biopython	1.85	Protein physicochemical property assessment (for example, calculate molecular weight)
	pandas	2.2.3	Calculation
	primer3	2.1.0	Design primers and primer analysis
	pyfaidx	0.8.1.3	Sequence extraction
	scipy	1.15.2	Calculation
	sklearn	1.6.1	Calculation
	statsmodels	1.6.1	Calculation
**Visualization**	cartopy	0.24.1	Drawing geographic map
	ete3	3.1.3	Drawing phylogenetic tree and domains
	networkx	3.4.2	Building networks
	pycirclize	1.9.0	Drawing Circos plot
**Frameworks**	PyQt5	5.15.9	Developing SPDE interface
	qfluentwidgets	1.7.6	Interface beautification
**Software**	clustalw2 (Thompson et al., 2003)	2.1	Sequence alignment
	diamond (Buchfink et al., 2015)	2.0.9.147	Sequence alignment
	mafft (Katoh et al., 2005)	7.037b	Sequence alignment
	muscle (Edgar, 2004)	3.8.31	Sequence alignment
	seqmap (Jiang and Wong, 2008)	1.0.13	Sequence alignment
	hmmbuild (Eddy, 2011)	3.3.2	Build hmm models
	hmmsearch (Eddy, 2011)	3.3.2	Identify gene family members

References

Buchfink, B., Xie, C., and Huson, D.H. (2015). Fast and sensitive protein alignment using DIAMOND. Nature Methods 12, 59-60.

Eddy SR (2011) Accelerated Profile HMM Searches. PLOS Computational Biology 7(10): e1002195

Edgar, R.C. (2004). MUSCLE: multiple sequence alignment with high accuracy and high throughput. Nucleic Acids Research 32, 1792-1797.

Jiang, H., Wong, W.H. (2008) SeqMap: Mapping Massive Amount of Oligonucleotides to the Genome. Bioinformatics, 24(20).

Katoh, K., Kuma, K.-i., Toh, H., and Miyata, T. (2005). MAFFT version 5: improvement in accuracy of multiple sequence alignment. Nucleic Acids Research 33, 511-518.

Thompson, J.D., Gibson, T.J., and Higgins, D.G. (2003). Multiple Sequence Alignment Using ClustalW and ClustalX. Current Protocols in Bioinformatics 00, 2.3.1-2.3.22.

Supplementary Table S4 Sequence data and the relevant NCBI accession IDs

ABC		ARF	MADS	NADPH		WRKY
NP_193656.2	NP_001329357.1	NP_180942.1	NP_001389198.1	WP_222905755.1	WP_079446045.1	XP_052159652.1
NP_194472.3	NP_194326.2	NP_568400.2	NP_001404294.1	WP_011700185.1	WP_281997562.1	NP_001409693.1
NP_190919.1	NP_001319112.1	NP_173414.1	NP_001390595.1	WP_367775786.1	WP_095645527.1	NP_001389276.1
NP_001189973.1	NP_001319754.1	NP_001233765.1	NP_001388994.1	WP_230378295.1	WP_169120394.1	NP_001393276.1
NP_195072.2	NP_001320481.1	NP_565161.1	NP_001388850.1	WP_050098317.1	WP_039646247.1	NP_001409692.1
NP_189475.1	NP_188746.2	NP_001031208.1	NP_179033.1	WP_011766694.1	WP_149616747.1	NP_001396287.1
NP_191473.2	NP_190363.3	NP_173356.1	NP_001342711.1	WP_104414600.1	WP_050422833.1	XP_048555655.1
NP_001327192.1	NP_001319673.1	NP_567841.1	NP_176712.1	WP_238633835.1	WP_048141317.1	NP_199447.1
NP_175561.1	NP_178241.1	NP_001190591.1	NP_001409189.1	WP_213740607.1	WP_049243480.1	NP_001234773.2
NP_174122.1	NP_001325068.1	NP_567119.1	NP_001391584.1	WP_316179523.1	WP_010889473.1	NP_001234802.2
NP_191092.1	NP_194275.2	NP_198518.1	NP_001407188.1	WP_211476592.1	WP_014500285.1	NP_174279.1
NP_175837.2	NP_001319729.1	KAG7642372.1	NP_001407446.1	WP_011939373.1		NP_567127.1
NP_175557.1	NP_850354.2	NP_001031706.1	NP_001411501.1	WP_079648672.1		NP_564792.1
NP_174115.1	NP_176961.1	NP_001031115.1	NP_001389915.1	WP_153526670.1		NP_181606.1
NP_173226.2	NP_176636.1	NP_001415187.1	NP_001388897.1	WP_067342714.1		NP_181263.2
NP_189477.4	NP_180259.1	NP_001391394.1	NP_176709.2	WP_114215390.1		NP_192354.1
NP_191575.2	NP_181467.1	NP_001409577.1	NP_001389342.1	WP_305278729.1		NP_193551.1
NP_175734.1	NP_176196.1	NP_001031548.1	NP_001408832.1	WP_282889009.1		NP_182248.1
NP_189480.1	NP_173005.1	NP_001331788.1	NP_596507.1	WP_424839464.1		NP_192034.1
NP_001327731.1	NP_001318302.1	NP_001396260.1	NP_001409733.1	WP_332118507.1		NP_567882.1
NP_001330984.1	NP_001323940.1	NP_001411795.1	NP_001388955.1	WP_012224814.1		NP_178565.1
NP_001319944.1	NP_188762.3	NP_001408638.1	KAF2954044.1	WP_350397279.1		NP_001352740.1
NP_181179.2	NP_175745.4	NP_001406599.1	Os01g0883100	WP_426030444.1		DAA05070.1
NP_001318859.1	NP_564383.1	NP_001408804.1	NP_001390992.1	WP_012332235.1		XP_052154622.1
NP_567813.1	NP_190916.1	NP_001407633.1	NP_001408119.1	WP_091786857.1		NP_001105837.1
NP_187973.1	NP_181228.1	NP_001406873.1	NP_001390263.1	WP_012384913.1		XP_008387690.2
At4g15233	NP_001031116.1	NP_001410552.1	NP_001410439.1	WP_205973235.1		XP_044397582.1
NP_200887.1	NP_001189675.1	NP_001391659.1	NP_001410290.1	WP_025200832.1		NP_001304498.2
NP_191069.2	NP_171754.1	NP_001406282.1	NP_001408621.1	WP_091810086.1		XP_044411874.1
NP_178984.1	NP_001332171.1	Os04g0664400	NP_001408325.1	WP_012468201.1		XP_008383508.3
NP_181272.1	NP_001327229.1	NP_001395946.1	NP_001403623.1	WP_414533302.1		XP_028954969.1
NP_196011.1	NP_201289.1	NP_001395963.1	NP_001393380.1	WP_012536148.1		XP_008343174.1
NP_187928.2	NP_187916.3	NP_001388650.1	NP_001389200.1	WP_377358474.1		XP_008381435.2
NP_190357.2	NP_194305.1	NP_001408668.1	KAB8108618.1	WP_009337322.1		
NP_194591.1	NP_180555.2	NP_001403679.1	NP_001406133.1	WP_012592192.1		
NP_192091.1	NP_193539.6	NP_001403601.1	NP_001392375.1	WP_291871756.1		
NP_190358.3	NP_198720.2	NP_001396727.1	Q84NC2.1	WP_075852376.1		
NP_563694.1	NP_001318860.1	NP_001411215.1	XP_025876460.1	WP_012648365.1		
NP_196555.2	NP_680694.3	NP_001411179.1	LOC543103	WP_019373987.1		
NP_001190972.1	NP_683599.1	NP_001409993.1	NP_001388916.1	WP_039800750.1		
NP_196862.1	NP_187917.3	NP_174701.2		WP_064240316.1		
NP_200635.1	NP_195847.1	NP_174699.2		WP_188027685.1		
NP_172973.1	NP_200098.1	Q9FX25.3		WP_012749792.1		
NP_192092.1	NP_196914.1	NC_003070.9		WP_010877850.1		
NP_190362.2	NP_199224.1	NC_003076.8		WP_048124788.1		
NP_176516.1	NP_566543.1	NC_090807.1		WP_044405275.1		
NP_182301.1	NP_565030.1			WP_011629021.1		
NP_191073.1	NP_189479.1			WP_011580680.1		
NP_001323911.1	NP_199466.1			WP_008644303.1		
KAL9291218.1	NP_200882.4			WP_145634075.1		
At5g06530	NP_194759.1			WP_331172137.1		
NP_191829.1	NP_189528.1			WP_011416265.1		
NP_176867.2	NP_001322407.1			WP_200292995.1		
NP_191071.1	CAD5316852.1			WP_135795694.1		
At1g04120	NP_197442.2			WP_246920655.1		
At1g30410	NP_001319703.1			WP_214184927.1		
At2g39480	NP_001326002.1			WP_011330958.1		
