# Supplementary material for: SPDEv3.0: A multidisciplinary integrated data analysis platform
Source: Plant Physiol. 2025 Oct 23;199(3):kiaf537. doi: 10.1093/plphys/kiaf537 (PMC12596362; doi:10.1093/plphys/kiaf537)
Supplement: kiaf537_Supplementary_Data [file kiaf537_supplementary_data.zip › Supplementary Video Legends.docx]

**SPDEv3.0: A Multidisciplinary Integrated Data Analysis Platform**

Dong Xu^1,2#*^, Kangming Jin^3*^, Quanling Zhang^4*^, Xianjia Zhao^5*^, Yanchun Li^4*^, Tingkai Wu^1,2^, Xiaobo Wang^1,2^, Yuan Yuan^1,2^, Zewei An^1,2^, Zhi Deng^1,2^, Wenguan Wu^1,2^, Han Cheng^1,2#^

*^1^National Key Laboratory for Tropical Crop Breeding, Rubber Research Institute, Chinese Academy of Tropical Agricultural Sciences, Haikou 571101, Hainan China*

*^2^Sanya Research Institute, Chinese Academy of Tropical Agricultural Sciences, Sanya 572024, Hainan China*

*^3^State Key Laboratory of Plant Environmental Resilience, College of Life Sciences, Zhejiang University, Hangzhou 310058, China*

*^4^Shenzhen Branch, Guangdong Laboratory of Lingnan Modern Agriculture, Genome Analysis Laboratory of the Ministry of Agriculture and Rural Affairs, Agricultural Genomics Institute at Shenzhen, Chinese Academy of Agricultural Sciences, Shenzhen 518120, China*

*^5^Bio-X Institutes, Key Laboratory for the Genetics of Developmental and Neuropsychiatric Disorders, Ministry of Education, Shanghai Jiao Tong University, Shanghai 200240, China*

*These authors contributed equally to this work.

#Correspondence: Han Cheng, Email: forcheng@gmail.com; Dong Xu, Email: xudongzhuanyong@163.com

Supplementary Video S1. Collinearity analysis between *A. thaliana* and *A. halleri*.

Supplementary Video S2. Identification and structural analysis of the maize *ARF* gene family.

Supplementary Video S3. Batch design process of SPDEv3.0 primers.

Supplementary Video S4. Batch CDS extraction process of SPDEv3.0.

Supplementary Video S5. Design process of full-length gene primers in SPDEv3.0.
